# Kinetic modelling of catalytic N_2_O removal

**DOI:** 10.1038/s41598-025-28647-6

**Published:** 2025-12-29

**Authors:** Mathias Nygård, Johan Wärnå, Vincenzo Russo, Jan Torrkulla, Dmitry Yu. Murzin

**Affiliations:** 1 Åbo Akademi University, Henriksgatan 2, Åbo, 20500 Finland; 2 Wärtsilä Finland Oy, Vaasa, 65101 Finland; 3 Università di Napoli ‘Federico II’, Chemical Sciences, Napoli, 80126 Italy

**Keywords:** Selective catalytic reduction, N_2_O catalyst, Emission aftertreatment, Ammonia-fuelled engine, Kinetic modelling, Energy science and technology, Chemistry

## Abstract

With decarbonisation of the marine industry, the use of hydrogen-based fuels such as ammonia introduces a need for development of the emission aftertreatment systems, for internal combustion engines. The main components are NO, NO_2_ and N_2_O formed in the combustion process, as well as ammonia that passes through the engine unburned. These products, alongside the altered emission profiles, pose a significant challenge for the emission aftertreatment, with the focus placed mainly on the development of the SCR system. Modelling of the catalytic removal of N_2_O from the exhaust gas was done as this component has the largest relative environmental impact of all the emissions from ammonia combustion. The studies have been aimed at gaining understanding of the factors that impact formation and removal of N_2_O in the aftertreatment system. Modelling of the experimental data has been done using a mechanistic model based on the surface reactions. The model was validated using the test data from catalyst screening in lab reactors.

**Supplementary Information:**

The online version contains supplementary material available at 10.1038/s41598-025-28647-6.

## Introduction

With sustainability and climate change becoming the most important concerns of the 21st century, the demand for sustainable solutions and technologies is rapidly increasing. The European Union has set goals for the decades to come regarding sustainability and carbon emissions, with net-zero greenhouse gas emissions to be achieved before the year 2050. With this also comes a need to renew the infrastructure and ecosystem of the energy business and the shipping sector. A major part of this sustainable transition is the introduction of green hydrogen-based fuels which can be produced with little or no carbon emissions, also called the decarbonisation of operations. Of these, ammonia has been proposed as one of the most promising fuels for the shipping sector where combustion engines are expected to remain the only viable option for long-distance transportation. This is because the shipping sector is a challenging area to introduce hydrogen-based fuels as the volume, storage needs, and transportability of the fuel strongly impact viability of the fuel selected for future marine installations.

The use of ammonia as a marine fuel introduces a few obstacles, of which safety and infrastructure are some of the most widely acknowledged hurdles to overcome. Less discussed, but equally important, is the need for an effective aftertreatment system that enables the ammonia-fuelled engine to positively affect the well-to-wake emissions of marine transportation. To remove the nitrogen-based emissions of an ammonia engine, selective catalytic reduction and oxidation have been proposed as viable solutions to both existing emissions, such as nitrogen oxides, and the newly introduced emissions of unburnt ammonia from the engine and the formed nitrous oxide. These new emissions will require new types of catalysts and a combination of different catalyst types in the optimal configurations to work as intended, which has been identified as a major area of research.

Burning ammonia in the fuel mix of an internal combustion engine, ICE, removes most of the carbon emissions and is in theory emission-free. The ideal combustion produces only water and nitrogen.

1 $$\:4N{H}_{3}+3{O}_{2}\to\:2{N}_{2}+6{H}_{2}O$$

**Fig. 1:**
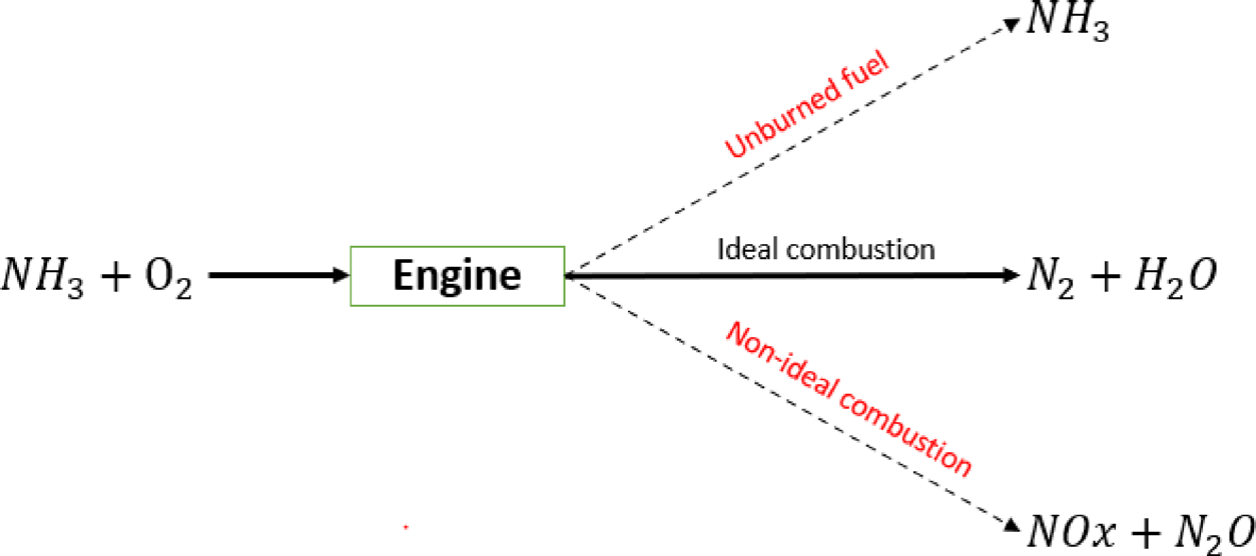
Illustration of ammonia combustion in internal combustion engines.

However, reaching the ideal stoichiometric combustion is not realistic and the real combustion can, thus, produce significant levels of NOx and varying levels of unburned ammonia in the exhaust stream (Fig. 1). Additionally, it introduces N_2_O as the main component with a high greenhouse gas potential in the absence of CO_2_.

Even though N_2_O has been a component of combustion engines for a long time, the amount of N_2_O is expected to increase for an ammonia-fuelled engine. Furthermore, as the main aim of the ammonia-fuelled engine concept is to reduce GHG emissions, the produced N_2_O will pose a significant drawback if not handled by the aftertreatment system^1^. The specifications of the ammonia-engine aftertreatment system are highly dependent on the engine tuning and combustion process, which alters the emission profile entering the aftertreatment system.

N_2_O has been described as one of the top contributors to human-related damage to the ozone layer and has a GHG potential that is 300 times larger than CO_2_ and is one of the most potent GHG, following closely after CO_2_ and methane which has a CO_2_-equivalent of about 25, calculated over 100 years^2^.

The purpose of the N_2_O removal catalyst is to manage the N_2_O formed in the combustion process since N_2_O has a high GHG potential and would increase the environmental impact of the ammonia-fuelled engine. Historically, N_2_O has not been removed from the internal combustion engine exhaust stream, because the formed concentrations with traditional fuels have been relatively low. This has led to the technology for catalytic abatement of N_2_O being in its infancy for low-temperature applications. The commercially available catalysts for N_2_O removal are aimed at the flue gas from nitric acid production plants and, therefore, they are most active at relatively high temperatures, compared to the exhaust streams from internal combustion engines^3^. The catalysts can directly decompose N_2_O into N_2_ and O_2_ according to Eq. (2), this reaction is slow at low temperatures and will, thus, have a marginal impact on the total N_2_O conversion. The catalysts can reduce N_2_O in the presence of a reducing agent such as propane or ammonia at much lower temperatures, according to Eq. (3).

2 $$\:2\:{N}_{2}O\to\:2\:{N}_{2}+{O}_{2}$$

3 $$\:3\:{N}_{2}O+2\:N{H}_{3}\:\to\:4\:{N}_{2}+3\:{H}_{2}O$$

As the SCR system already has the functionality for dosing ammonia in the form of urea, and with the engine exhaust also containing ammonia, this is the preferred reagent in this application, thus allowing for both a passive and active reagent flow to the catalyst. With ammonia being available for engine combustion, the perspective of using direct ammonia injection as a reducing agent is an interesting possibility for the emission aftertreatment system. Ability of a catalyst to directly decompose N_2_O or reduce N_2_O with a reagent, depends on the catalyst, nature and concentration of the reducing agent, temperature, and other factors. Presence of oxygen, water and nitric oxide can influence the overall catalytic performance and its activity towards removal of N_2_O^4–7^.

Iron-exchanged zeolites have been extensively studied as possible N_2_O removal catalysts in the past decade. The high durability and stability of these materials, along with their relatively low costs, are the reasons they are commonly referred to as the most plausible catalysts. However, several key features determine the effectiveness of the catalyst, of which the N_2_O conversion capability and N_2_O selectivity are the most important. These two factors are very temperature dependent and finding a catalyst that has a high conversion and a high N_2_O selectivity at relatively low temperatures is what defines which type of catalyst is the most promising.

Iron zeolites using β, ferrierite or ZSM-5 zeolites are most often studied. For N_2_O decomposition, the zeolites with pores slightly larger than the N_2_O molecule are proposed to be the most active ones. Iron-ferrierite is in this range with the pore openings of ca. 7-7.5 Å. The boost in activity is often ascribed to the mechanism where two active sites can interact with the same N_2_O molecule, thereby decomposing it more effectively. This does not, however, mean that Iron-ferrierite is a superior catalyst for N_2_O removal as the activity increase is only reported in N_2_O decomposition in the absence of nitrogen oxide^8^.

Most zeolites are also selective and active in reducing nitrogen oxides, such as NO and NO_2_, in a standard NH_3_-SCR. Reduction of N_2_O is, therefore, also dependent on the presence of other reducible species in the exhaust flow through the catalyst. Thus, simultaneous reduction of NOx and N_2_O has been studied as it was thought that N_2_O reduction would be inhibited by the standard SCR reaction of NO^6^, which at a lower activation energy would take place before reduction of N_2_O. This was, however, not the case, and it was found that the N_2_O removal over Fe-BEA was not inhibited by the presence of nitrogen oxides. On the contrary, the presence of NO was found to increase N_2_O conversion. Therefore, a stoichiometric equation for the overall NO-assisted N_2_O-reduction in the presence of NH_3_ proceeds^6^ according to (4).

4 $$\:2\:NO+{N}_{2}O+2\:N{H}_{3}\:\to\:3\:{N}_{2}+3\:{H}_{2}O$$

The presence of nitrogen oxides was, therefore, concluded to lower the light-off temperature for the N_2_O-reduction, and the NO-reduction was also observed to increase in the presence of N_2_O. This strengthens the conclusion that the two must have a synergistic reaction. However, increasing the NO/N_2_O ratio did have an impact on the N_2_O conversion, with higher ratios corresponding to a marginally lower conversion^6^. Another interesting finding in the study^6^ was that N_2_O reduction was hampered by a large NH_3_ surplus, which was not the case for the NO reduction. This seemed to indicate that N_2_O reduction came to a point of saturation as the NH_3_ feed was increased and further increase caused inhibition of the active sites for N_2_O reduction.

For N_2_O decomposition, the presence of nitrogen oxide was found to elevate N_2_O conversion up to a NO/N_2_O ratio of 0.25 which saturated the N_2_O decomposition activity, and no further improvement was observed at higher ratios^4^.

Additionally, no inhibition was observed for NO/N_2_O ratios up to 10, indicating that NO assistance is only beneficial for N_2_O decomposition. From this, a NO-assisted mechanism for N_2_O decomposition can be suggested as in (5)^4^.

5 $$\:NO+{N}_{2}O\:\to\:\:{N}_{2}+N{O}_{2}$$

### Model development

Modelling the emission aftertreatment system based on the available data and the literature provides an opportunity to see how the catalyst would work under simulated conditions in areas where full-scale testing has not been carried out or is expected to be challenging, such as a low-temperature operation. The model needs to be dependent on such parameters that can be measured in the field, and preferably avoid all parameters that are not available in the full-scale system to allow for comparison in the simulated efficiency and the actual efficiency of the system under known conditions. This, however, will mean that parameters that would allow for higher accuracy of the model cannot be used.

Kinetic models are often tailored to one catalyst type and the included reactions may vary depending on the catalyst type, while also a model for one catalyst type can be used to describe other catalysts, if the reactions are similar. This cannot be done without studying validity of the model for the catalyst in question. This model will assume that the available test data are from the Fe-zeolite catalyst, or similar variants, thus allowing, for the reaction routes described in the literature for such catalyst types.

Adapting a kinetic model for the N_2_O catalyst requires some understanding of the reactions taking place on the surface of the catalyst. This includes adsorption and desorption as well as the chemical reactions between components in the adsorbed layer. A more thorough understanding of the catalyst specific reactions will allow for the construction of a more accurate model. Furthermore, the reactions will, in most cases, inclusion of the temperature dependence.

**Fig. 2:**
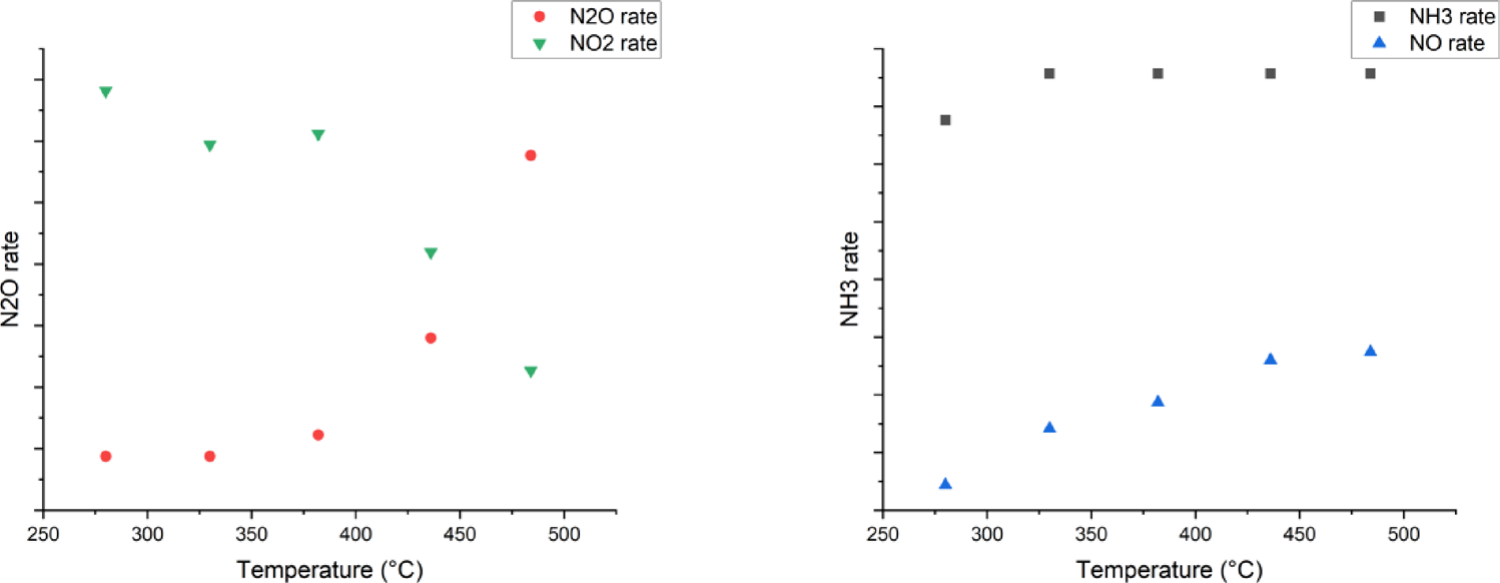
Temperature dependencies of the reaction rates. Propriety data set.

The need for temperature dependent models described with the Arrhenius equation was observed in the data from a lab reactor testing over a broad temperature range using a catalyst bed of ca. 5 cm (Fig. 2). An increase in N_2_O rate of removal with temperature was seen as expected, while the removal rates for NH_3_ and NO consumption were seen to be less affected by increasing temperatures. This can be contributed to the adsorption constants of these components being heavily temperature dependent. As the adsorption equilibrium constants are negatively affected by higher temperatures, due to the exothermic nature of adsorption, the overall increase in the consumption rate is, thus, counteracted by the decrease in adsorption. This behaviour can also be the cause of the decrease in the rate of NO_2_ consumption with temperature although this can also be ascribed to other factors, such as NO_2_ formation from NO-assisted N_2_O decomposition, as reported by Pérez-Ramírez et al.^4^.

**Scheme 1:**
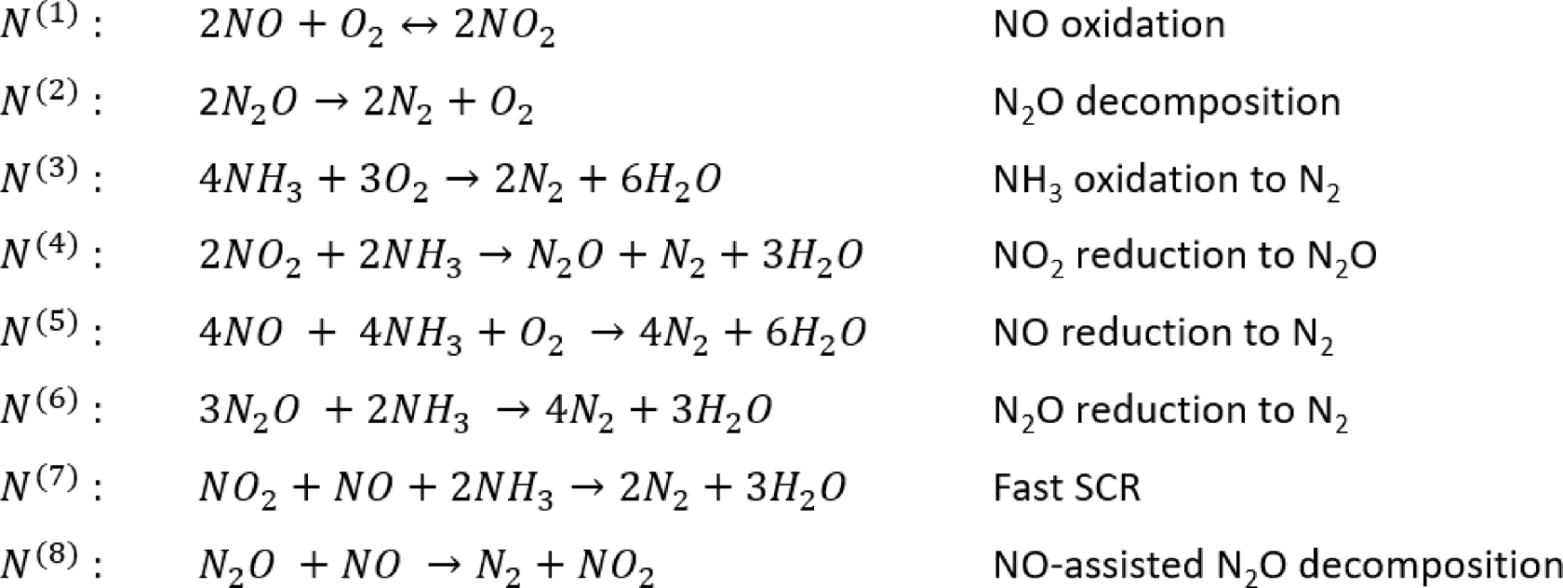
Reaction routes of the kinetic model.

There are several main reaction routes described as present on the Fe-zeolite N_2_O catalyst. Of these, eight routes have been assumed to take place on the surface of the catalyst to a such extent that they impact the overall concentrations. These are the oxidation reaction of NO^9^, decomposition reaction of N_2_O^6^ and the reduction reactions of NO, NO_2_^10^ and N_2_O^11^. Furthermore, the undesired reactions of NH_3_ oxidation and N_2_O formation^5^ are assumed to be present to explain deviations in NH_3_ consumption over the catalyst and the possible N_2_O formations at lower temperatures. These main routes are visible in Scheme 1 along with the assigned main route numbers N^(1)^ - N^(8)^, which will be used for further identification of the main routes. In the development of the model, partial oxidation of ammonia to N_2_O and NO was not considered.

**Fig. 3:**
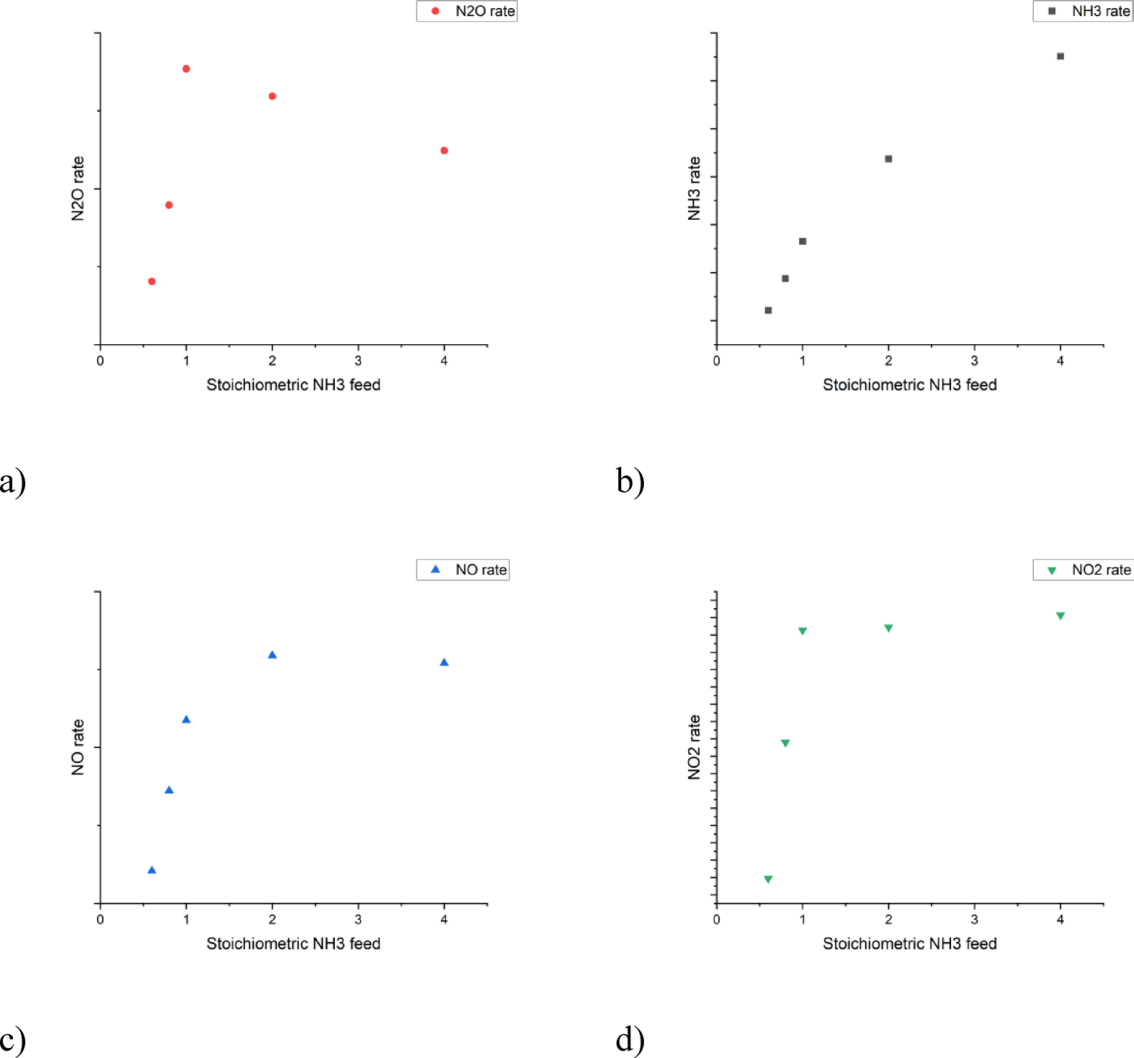
Influence of the ammonia excess on catalytic data.

Reduction using ammonia as a reagent in basic routes N^(4)^- N^(7)^, was assumed to be present due to an increase in the reaction rates for reduced components (e.g. dinitrogen and N_2_O), when increasing the stoichiometric fraction of NH_3_ in the feed (Fig. 3). The reduced components are N_2_O, NO and NO_2_. The reaction rates were found to increase up to a stoichiometric NH_3_ feed composition of 1.0, which implies that there is enough ammonia present to completely reduce all reducible components in the feed gas. Further increasing the feed of ammonia over the stoichiometric feed composition of 1.0 (Fig. 3) induces an NH_3_ surplus, which was found to increase the rates only marginally, or decrease the rates of consumption, indicating that there is an inhibiting effect of the ammonia surplus. The rate of NO_2_ consumption was also observed to be negative in a stoichiometric NH_3_ feed below 1.0, indicating that there is more NO_2_ formed from routes N^(1)^ and N^(8)^, than NO_2_ used in reduction routes N^(4)^ and N^(7)^. The undesired NH_3_ oxidation was observed from the tests with the stoichiometric surplus of ammonia.

### Thermodynamic analysis

The equilibrium composition of N_2_O removal reaction network from Scheme 1 was computed using the Gibbs reactor module implemented in ChemCAD software^12^. The non-ideality of the system was taken into account adopting the Soave-Redlich-Kwong equation of state, as suggested in the literature^13^. The computations were conducted fixing a total pressure of 1 bar, in a temperature range between 50 and 800 °C, and the following feed composition: NH_3_ -1 mol, NO-0.95 mol, NO_2_ – 0.05 mol, N_2_O- 0.05 mol, H_2_O − 100 mol, O_2_- 50 mol, N_2_- 848 mol, in accordance with the experiments.

**Fig. 4:**
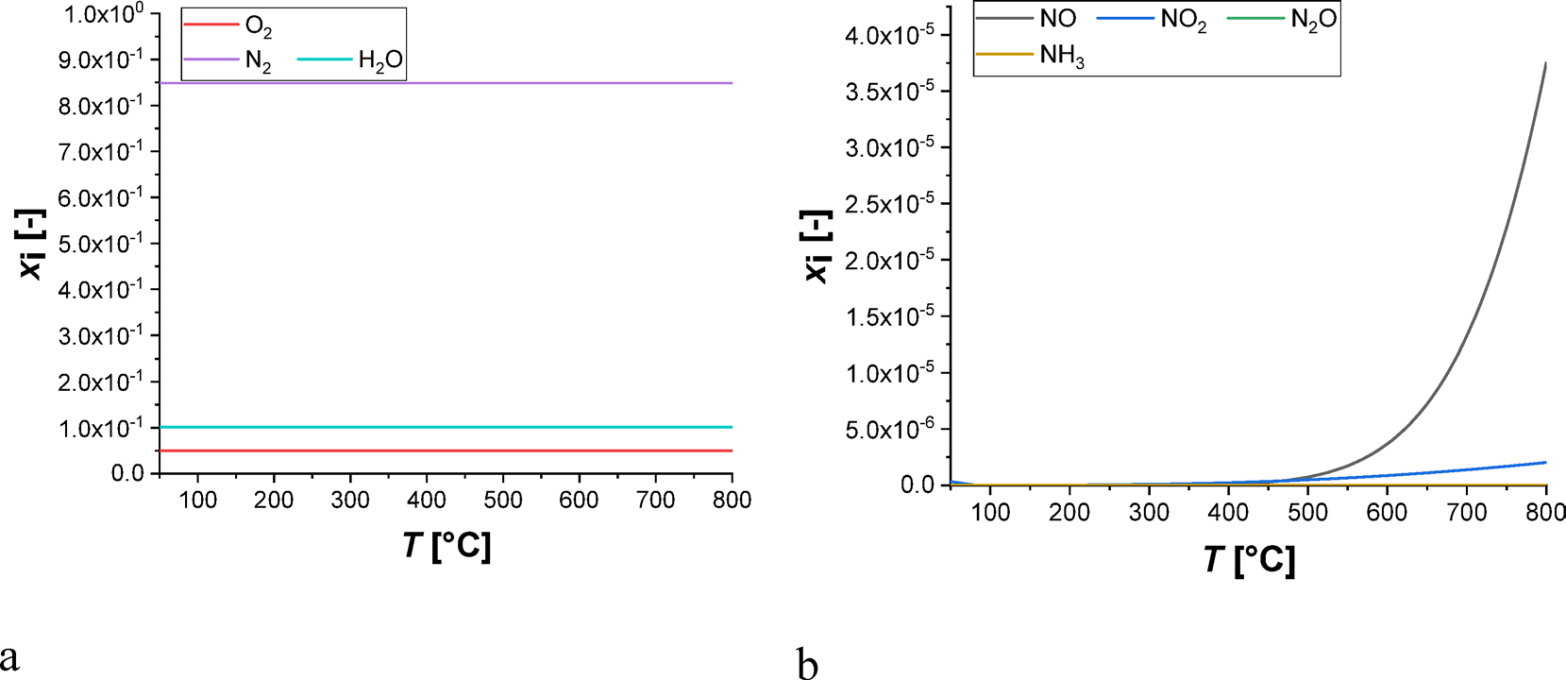
Equilibrium composition of the reaction mixture as a function of temperature.

The calculated composition, in terms of molar fractions *x*_*i*_, is reported in Fig. 4, revealing that as expected from the thermodynamic viewpoint nitrogen, oxygen and water are the main components in the whole temperature range.

### Detailed reaction mechanism

The main routes are not suitable for kinetic modelling directly based on only formal kinetics, as they comprise several elementary reactions, including adsorption or desorption on the active sites and formation of intermediate products. A good example of this is the basic route one, N^(1)^, that contain competitive adsorption of NO and O_2_, oxidation of NO to NO_2_ and desorption of NO_2_.

The full list of all elementary reactions, and respective stoichiometric coefficients or so-called Horiuti numbers^14,15^ needed to balance the components for each basic route, is available in Table 1. This table contains the main elementary steps, which are reported in the literature for the routes in Scheme 1. It should be noted that steps with the sign $\:\equiv\:$ are considered to be quasi-equilibrated (i.e. fast in both directions). Several steps with the sign $\:\to\:$ are irreversible, thus substantially simplifying derivation of the rate equations.

In the Table 1 the asterisk, $\:*$, corresponds to the active sites on the surface of a catalyst and components followed by an asterisk are adsorbed on the surface of the catalyst. Note that depending on the catalyst the elementary steps could be more complicated. For example, dissociation of dioxygen can require binuclear iron sites.

**Table 1:** The main independent routes comprising elementary reactions*.

Num	Elementary reaction	$\:{\boldsymbol{N}}^{\left(1\right)}$	$\:{\boldsymbol{N}}^{\left(2\right)}$	$\:{\boldsymbol{N}}^{\left(3\right)}$	$\:{\boldsymbol{N}}^{\left(4\right)}$	$\:{\boldsymbol{N}}^{\left(5\right)}$	$\:{\boldsymbol{N}}^{\left(6\right)}$	$\:{\boldsymbol{N}}^{\left(7\right)}$	$\:{\boldsymbol{N}}^{\left(8\right)}$
1	$\:NO+\:*\:\equiv\:NO*$	2	0	0	0	4	0	1	1
2	$\:{O}_{2}+2\:*\:\equiv\:2O*$	1	-1	3	0	1	0	0	0
3	$\:NO*+\:O\:*\:\leftrightarrow\:N{O}_{2}*\:+\:*$	2	0	0	2	0	0	1	-1
4	$\:N{O}_{2}\:\equiv\:N{O}_{2}+\:*$	-2	0	0	0	0	0	0	0
5	$\:{N}_{2}O+\:*\:\equiv\:{N}_{2}O*$	0	2	0	-1	0	3	0	1
6	$\:{N}_{2}O*\:\to\:{N}_{2}+O*$	0	2	0	1	0	1	0	0
7	$\:{NH}_{3}+\:*\:\equiv\:N{H}_{3}*$	0	0	4	2	4	2	2	0
8	$\:{NH}_{3}+\:*\:\equiv\:N{H}_{2}*+\:H*$	0	0	4	2	4	2	0	0
9	$\:N{H}_{2}*+\:*\:\equiv\:NH*+\:H*$	0	0	4	0	0	0	0	0
9’	$\:NH*\:+\:*\:\underrightarrow{Fast}\:N*\:+\:H*$	0	0	4	0	0	0	0	0
9’’	$\:2N*\:\:\underrightarrow{Fast}{N}_{2}+\:\:*$	0	0	2	0	0	1	0	0
9’’’	$\:O*+H\:*\:\:\underrightarrow{Fast}OH*\:+*$	0	0	6	1	2	1	0	0
9’’’’	$\:OH*+\:H*\:\:\underrightarrow{Fast}{H}_{2}O*\:+\:*$	0	0	6	1	2	1	0	0
10	$\:{NH}_{2}*+\:N{O}_{2}*\:\to\:{N}_{2}O*\:+\:{H}_{2}O*$	0	0	0	2	0	0	0	0
11	$\:N{H}_{2}*+\:{N}_{2}O\:*\:\to\:{N}_{2}+{H}_{2}O*\:+\:N*$	0	0	0	0	0	2	0	0
12	$\:NO*\:+\:{N}_{2}O*\:\to\:N{O}_{2}*+{\:N}_{2}+\:*$	0	0	0	0	0	0	0	1
13	$\:{H}_{2}O\:*\:\equiv\:{H}_{2}O+\:*$	0	0	6	3	6	3	3	0
14	$\:NO*+N{H}_{2}*\:\to\:{N}_{2}+{H}_{2}O*\:+\:*$	0	0	0	0	4	0	0	0
15	$\:{2NO}_{2}*+\:{H}_{2}O*\:\to\:HN{O}_{3}*\:+\:HONO*$	0	0	0	0	0	0	1	0
15’	$\:HN{O}_{3}*+\:NO*\:\:\underrightarrow{Fast}N{O}_{2}*\:+\:HONO*$	0	0	0	0	0	0	1	0
15’’	$\:{NH}_{3}+HONO*\:\:\underrightarrow{Fast}{N}_{2}+\:2{H}_{2}O*$	0	0	0	0	0	0	2	0

*The corresponding overall equations for the basic routes are given in Scheme 1.

Assuming that all but one of the elementary steps of each basic route are in quasi-equilibria, the reaction rate of each route is dependent on the rate of the so-called rate-limiting elementary step. This allows for simplifications of the whole system of reactions to a more manageable size. The reaction rates for all main routes can, thus, be solved as equal to the coverage dependent rate-limiting elementary steps.

6 $$\:{r}^{N\left(1\right)}={r}_{3}={r}_{+3}-{r}_{-3}={k}_{+3}{\theta\:}_{NO*}{\theta\:}_{O*}-{k}_{-3}{\theta\:}_{N{O}_{2}*}{\theta\:}_{v*}$$

7 $$\:{r}^{N\left(2\right)}={r}_{6}={r}_{+6}={k}_{+6}{\theta\:}_{{N}_{2}O*}$$

8 $$\:{r}^{N\left(3\right)}={r}_{9}={r}_{+9}={k}_{+9}{\theta\:}_{N{H}_{2}*}{\theta\:}_{v*}$$

9 $$\:{r}^{N\left(4\right)}={r}_{10}={r}_{+10}={k}_{+10}{\theta\:}_{{NO}_{2}*}{\theta\:}_{N{H}_{2}*}$$

10 $$\:{r}^{N\left(5\right)}={r}_{14}={r}_{+14}={k}_{+14}{\theta\:}_{NO*}{\theta\:}_{N{H}_{2}*}$$

11 $$\:{r}^{N\left(6\right)}={r}_{11}={r}_{+11}={k}_{+11}{\theta\:}_{{N}_{2}O*}{\theta\:}_{N{H}_{2}*}$$

12 $$\:{r}^{N\left(7\right)}={r}_{15}={r}_{+15}={k}_{+15}{\theta\:}_{N{O}_{2}*}{\theta\:}_{{H}_{2}O*}$$

13 $$\:{r}^{N\left(8\right)}={r}_{12}={r}_{+12}={k}_{+12}{\theta\:}_{{N}_{2}O*}{\theta\:}_{NO*}$$

The reaction rates are dependent on the coverages of adsorbed species on the surface of the catalyst. The coverage of species that can also be found in the gas phase are simply calculated with the adsorption coefficients times the partial pressures of the gaseous species, assuming molecular adsorption without dissociation. The only exception is dissociative adsorption of oxygen.

14 $$\:{\theta\:}_{O*}=\:\sqrt{{K}_{{O}_{2}}{P}_{{O}_{2}}{\theta\:}_{\upsilon\:*}}$$

15 $$\:{\theta\:}_{NO*}={K}_{NO}{P}_{NO}{\theta\:}_{\upsilon\:*}\:$$

16 $$\:{\theta\:}_{{NO}_{2}*}={K}_{N{O}_{2}}{P}_{N{O}_{2}}{\theta\:}_{\upsilon\:*}\:$$

17 $$\:{\theta\:}_{{N}_{2}O*}={K}_{{N}_{2}O}{P}_{{N}_{2}O}{\theta\:}_{\upsilon\:*}\:$$

18 $$\:{\theta\:}_{N{H}_{3}*}={K}_{N{H}_{3}}{P}_{N{H}_{3}}{\theta\:}_{\upsilon\:*}\:$$

19 $$\:{\theta\:}_{{H}_{2}O*}={K}_{{H}_{2}O}{P}_{{H}_{2}O}{\theta\:}_{\upsilon\:*}\:$$

With the assumptions that reaction 9’-9’’’’, 15’ and 15’’ are all very fast, it can be concluded that coverages of $\:H*$, $\:NH*$, $\:N*$, $\:OH*$, $\:HN{O}_{3}*$ and $\:HONO*\:\:$are inferior, as they will be consumed rapidly as soon as they are generated. This leaves the coverage of $\:N{H}_{2}*$ as the only coverage of a component not present in the gas phase that needs to be calculated. As this component does not appear in the gaseous phase, and as such is not measured, the coverage of this component is solved from the steady state approximation that concentration of $\:N{H}_{2}*$ is time independent. Subsequently, the generation rate of this adsorbed species is equal to the rate of consumption (20),

20 $$\:{r}_{8}={r}_{9}{+\:r}_{10}+\:{r}_{14}+{r}_{11}$$

giving (21).

21 $$\:{{k}_{+8}\theta\:}_{N{H}_{3}*}{\theta\:}_{v*}={{k}_{+9}\theta\:}_{N{H}_{2}*}{\theta\:}_{v*}+{k}_{+10}{\theta\:}_{N{H}_{2}*}{\theta\:}_{{NO}_{2}*}+{k}_{+14}{\theta\:}_{N{H}_{2}*}{\theta\:}_{NO*}+{k}_{+11}{\theta\:}_{N{H}_{2}*}{\theta\:}_{{N}_{2}O*}$$

In (21) the only unknown is the coverage of $\:N{H}_{2}*$, leading to (22).

22 $$\:{\theta\:}_{N{H}_{2}*}=\frac{{k}_{+8}{\theta\:}_{N{H}_{3}*}{\theta\:}_{v*}}{{k}_{+9}{\theta\:}_{v*}{+\:k}_{+10}{\theta\:}_{{NO}_{2}*}+{k}_{+14}{\theta\:}_{NO*}+{k}_{+11}{\theta\:}_{{N}_{2}O*}}$$

Further replacing the coverages with the partial pressures and adsorption coefficients a tractable expression for the $\:N{H}_{2}*$ coverage is obtained.

23 $$\:{\theta\:}_{N{H}_{2}*}=\frac{{k}_{+8}{K}_{{NH}_{3}}{P}_{{NH}_{3}}\text{}{\theta\:}_{v*}}{{k}_{+9}{+\:k}_{+10}{K}_{N{O}_{2}}{P}_{N{O}_{2}}+{k}_{+14}{K}_{NO}{P}_{NO}+{k}_{+11}{K}_{{N}_{2}O}{P}_{{N}_{2}O}}$$

Finally, when all coverages are expressed through the fraction of vacant sites, $\:{\theta\:}_{v*}$, the latter can be computed from the balance equation for the coverages.

24 $$\:\sum\:{\theta\:}_{i}=\:{\theta\:}_{v*}+{\theta\:}_{O*}+{\theta\:}_{NO*}+{\theta\:}_{N{O}_{2}*}+{\theta\:}_{{N}_{2}O*}+{\theta\:}_{{NH}_{3}*}+{\theta\:}_{{H}_{2}O*}+{\theta\:}_{N{H}_{2}*}=1\:$$

The fraction of vacant sites is calculated by replacing the coverages with their dependencies on the partial pressures and adsorption coefficients, giving the final equation for the fraction of vacant sites:

25 $$\:{\theta\:}_{v*}=\frac{1}{1+\sqrt{{K}_{{O}_{2}}{P}_{{O}_{2}}}+{K}_{NO}{P}_{NO}+{K}_{N{O}_{2}}{P}_{N{O}_{2}}+{K}_{{N}_{2}O}{P}_{{N}_{2}O}+{K}_{N{H}_{3}}{P}_{N{H}_{3}}+{K}_{{H}_{2}O}{P}_{{H}_{2}O}+\frac{{k}_{+8}{K}_{{NH}_{3}}{P}_{{NH}_{3}}\text{}}{{k}_{+9}{+\:k}_{+10}{K}_{N{O}_{2}}{P}_{N{O}_{2}}+{k}_{+14}{K}_{NO}{P}_{NO}+{k}_{+11}{K}_{{N}_{2}O}{P}_{{N}_{2}O}}}\:$$

**Fig. 5:**
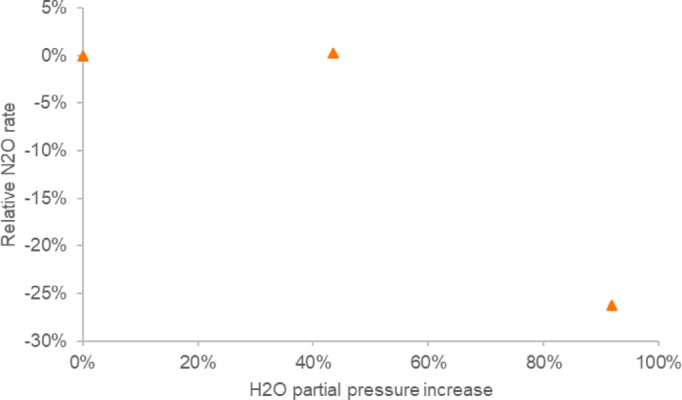
Inhibition of N_2_O consumption rate by H_2_O.

The fraction of vacant sites also describes the effect from inhibition by relating the coverages of all components to each other. The inhibition effect is, thus, caused by the coverage of some reactants being relatively large compared to the other coverages. This can be seen in Fig. 3, where an increasing surplus of ammonia causes the consumption rates of other components, such as N_2_O and NO, to decrease. This inhibition can also be from non-reactive coverages of components that are either present in the gas phase or formed in the reactions. In particular, H_2_O is known to have an inhibiting effect. The inhibiting effect of water on N_2_O consumption was observed for a test with increasing H_2_O partial pressures in the feed gas (Fig. 5). The inhibiting effect on N_2_O decomposition rate by H_2_O in the feed is also clearly documented in the literature^6,11,16^, while presence of oxygen did not influence the performance of iron-ferrierite catalyst^16^.

The importance of the inhibition by water in the exhaust gas becomes more apparent when considering that the content of water in the flue gas increases with ammonia combustion compared to conventional fuels, such as liquefied natural gas or light fuel oil.

At the initial stage of the model development, partial oxidation of ammonia to NO has been also incorporated, considering an additional step in the reaction mechanism. Namely adsorbed N_2_O was considered to react with adsorbed oxygen giving NO, which then desorbs from the surface. The data fitting with the results presented in Supporting Information illustrated the same fitting in terms of accuracy of the explanation of experimental data, therefore below only the results for the model from Table 1 will be presented.

To simplify determination of the constants, the Arrhenius equation is transformed into a form that removes the correlations between the pre-exponential factor, $\:A$, and the activation energy, $\:E{a}_{i}$, by introducing the average temperature of experiments, $\:{T}_{avg}$, and the rate at the average temperature, $\:{k}_{avg,\:\:i}$. This transforms the Arrhenius equation into the following form^17^:

26 $$\:{k}_{i}={{k}_{avg,\:\:i}\:e}^{\left(\frac{-E{a}_{i}}{R}\left(\frac{1}{T}-\frac{1}{{T}_{avg}}\right)\right)}$$

which is less likely to suffer from the correlations between the pre-exponential factor and the activation energy.

To estimate the unknown parameters a program was constructed in MATLAB, which consisted of an optimisation program coupled with an ODE solver using the plug flow reactor model to find the best estimation for predicting the actual measured data. This is done considering the concept of the least squares ( Eq. 27 ) but with an added weighting factor, $\:{w}_{i}$, for each component. This weighting factor allows for prioritisation of certain components in the exhaust stream.

27 $$\:min\sum\:{\left(\left({c}_{i,\:calc}-{c}_{i,\:meas}\right){w}_{i}\right)}^{2}$$

Another approach to the optimisation of the parameters was to utilise the least squares of the relative deviations (28). The relative deviation is expressed as the conversion of each component in percent. This removes prioritisation of the components with higher concentrations, which is the case with the absolute deviation. The weighting factor was still added as a possibility to further prioritise some components and to remove the dependence on abundant components that are magnitudes of order higher in concentration and measured at a lower accuracy, such as O_2_ and N_2_. Removing the dependency on O_2_, H_2_O, and N_2_ increased the accuracy on the main components while there was no observable impact on the accuracy of these components.

28 $$\:min\sum\:{\left(\left(\frac{{c}_{i,0}-{c}_{i,calc}}{{c}_{i,0}}-\frac{{c}_{i,0}-{c}_{i,meas}}{{c}_{i,0}}\right){w}_{i}\right)}^{2}$$

### Model validation

Estimating the parameters for the N_2_O catalyst rate constants was found to be challenging. The number of unknown parameters is 32, of which 16 are rate constants at the average temperature, $\:{k}_{0,\:\:i}$ and 16 are either the respective activation energies or heat of adsorption (Table 2). The large number of parameters introduced an issue related to determination of the global minimum, as the starting values had a large impact on the outcome of the calculations. This issue was observed on particular occasions, when the latest solution was used as the starting point with one or two parameters changed, resulting in another local minimum where most parameters had different values from the previous solution.

**Table 2:** Parameters in the kinetic N_2_O model.

Parameter	Name	Parameter	Name
Adsorption constant at average T, O_2_	K^avg^_O*_	H_O*_	Heat of adsorption, O_2_
Adsorption constant at average T, NO	K^avg^_NO*_	H_NO*_	Heat of adsorption, NO
Adsorption constant at average T, NO_2_	K^avg^_NO2*_	H_NO2*_	Heat of adsorption, NO_2_
Adsorption constant at average T, N_2_O	K^avg^, _N2O*_	H_N2O*_	Heat of adsorption, N_2_O
Adsorption constant at average T, NH_3_	K^avg^_NH3*_	H_NH3*_	Heat of adsorption, NH_3_
Adsorption constant at average T, H_2_O	K^avg^_H2O*_	H_H2O*_	Heat of adsorption, H_2_O
Reaction rate constant at average T, k_3_ forward	k^avg^_k3,f_	$\:{Ea}_{k3,f}$	Activation energy, k3 forward
Reaction rate constant at average T, k_3_ reverse	k^avg^_k3,rev_	$\:{Ea}_{k3,r}$	Activation energy, k3 reverse
Reaction rate constant at average T, k_6_	k^avg^_k6_	$\:{Ea}_{k6}$	Activation energy, k6
Reaction rate constant at average T, k_8_	k^avg^_k8_	$\:{Ea}_{k8}$	Activation energy, k8
Reaction rate constant at average T, k_9_	k^avg^_k9_	$\:{Ea}_{k9}$	Activation energy, k9
Reaction rate constant at average T, k_10_	k^avg^_k10_	$\:{Ea}_{k10}$	Activation energy, k10
Reaction rate constant at average T, k_11_	k^avg^_k11_	$\:{Ea}_{k11}$	Activation energy, k11
Reaction rate constant at average T, k_12_	k^avg^_k12_	$\:{Ea}_{k12}$	Activation energy, k12
Reaction rate constant at average T, k_14_	k^avg^_k14_	$\:{Ea}_{k14}$	Activation energy, k14
Reaction rate constant at average T, k_15_	k^avg^_k15_	$\:{Ea}_{k15}$	Activation energy, k15

Several starting points were initially tested, being derived from the determined parameter values in the literature and observed phenomena in the experimental data. These initial points produced superficially good solutions, being found afterwards unlikely, either by comparing the temperature dependencies with the literature, or with reaction rates of other reactions.

To increase the chances of the solver finding a good solution a few basic conditions were introduced as boundaries, including positive reaction rates. This included most of the rates where the elementary reactions generated either nitrogen or water. The same goes for most oxidation reactions, such as the undesired oxidation of ammonia. An exception to this rule is oxidation of NO to NO_2_, as for which in the basic route N^(1)^ NO_2_ can be reduced back to NO and dioxygen under certain conditions. This is the reason for the reverse reaction present in the rate for the overall reaction, as the total reaction rate for the corresponding route can be negative whilst the rates for both forward and reverse reactions are positive.

The adsorption coefficients are also set to be positive. The heat of adsorption is negative because adsorption exothermic. The reason for setting the boundaries was to have physically reasonable values of the parameters.

**Fig. 6:**
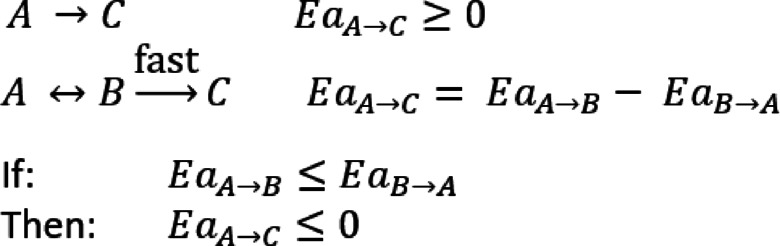
Visual representation of the apparent negative activation energies.

The activation energy for an elementary reaction should be positive, except for trimolecular reactions. This, however, was not applied to the model for the simple reason that the assumed reactions could be further dependent on several elementary steps that had not been identified. Assuming that one or more of these steps are reversible, and that the reverse reaction had a larger temperature dependence than the forward reaction, the outcome would be a net negative activation energy as visualised in Fig. 6.

**Fig. 7:**
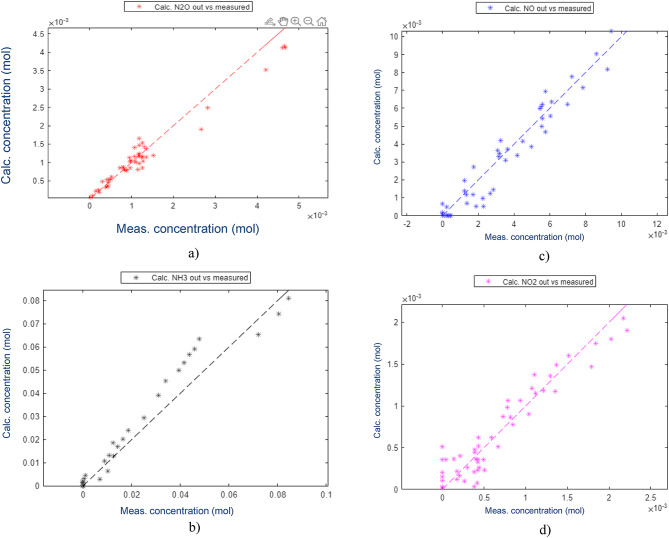
Estimated outlet concentrations vs. measured concentrations.

The kinetic model was found to be capable of describing the N_2_O behaviour, Fig. 6. The estimated outlet concentrations after the catalyst bed compared to the actual measured concentrations showed an adequate performance of the model for all components within the measurement accuracy of the test data. The components used in the estimation of the parameters were NH_3_, NO, NO_2_ and N_2_O (Fig. 7) while the other components were only controlled to validate that the model was indeed calculating them correctly. The other components are O_2_, N_2_ and H_2_O, which all have concentrations several magnitudes higher than the ones used for estimation as clear from the feed composition mentioned in the section on thermodynamic analysis.

This is partly the reason why other components were not used in the data fitting, as the absolute deviations would always favour the components with high concentrations. Additionally, these components are not measured accurately, and some are only calculated concentrations, as is the case with N_2_ used as the balance gas in the testing.

In these calculations three sets of test data from different samples were used and the parameter values were separately estimated for each catalyst.

It should be noted that an external provider was commissioned to run the tests, thus exact details about the catalyst manufacturer, specific loading and washcoat thickness are confidential. The lab reactor catalyst samples were cut out from monolithic catalyst blocks and were in the sizes of 17 ml, 20 ml and 30 ml for samples 1–3 respectively.

These samples were tested in a GHSV ranging from 15 10^3^ and 60 10^3^ h^− 1^.

**Fig. 8:**
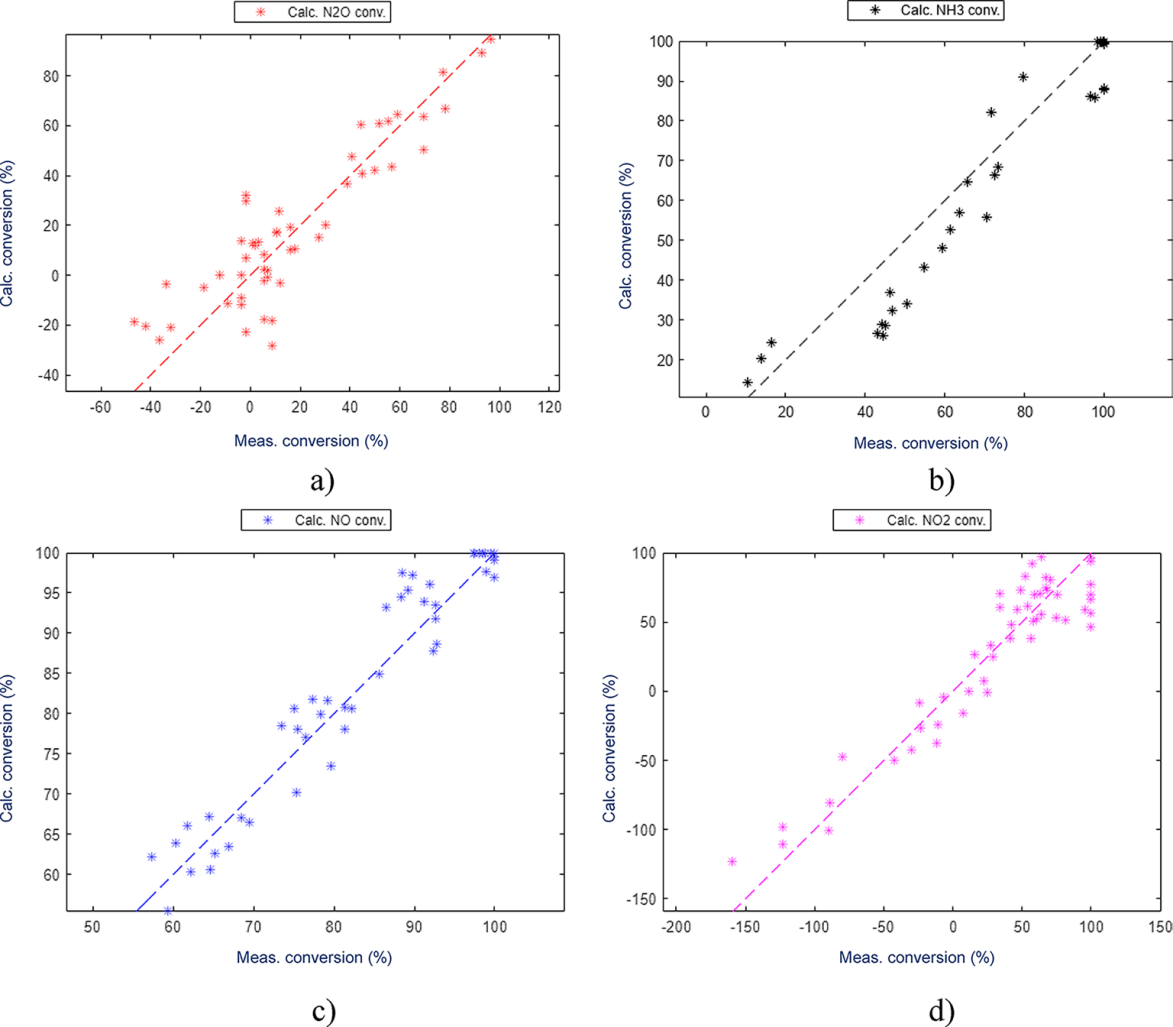
Estimated vs. measured conversions for catalyst formulation 1.

To simplify the discussion, catalyst 1 out of these tested will be more thoroughly discussed (Fig. 8). The test data was acquired as a part of a larger test campaign, where sourced catalyst samples from commercial catalyst manufactures were tested in a small lab scale reactor. The test campaign was carried out to determine the functionality of the catalyst samples in a specified set of operating conditions similar to what could be expected from a marine catalyst installation.

As the most important component for the correct estimation is N_2_O, the focus was put on having explained all possible phenomena that could affect this compound. The estimation was initially done with a higher weighting factor for N_2_O. This was found to provide a very good estimation of N_2_O while not explaining the other important components well enough. Once a good N_2_O estimation was found, this was used as a starting point for the estimation with similar weighting factors for all important components. This allowed for a minor increase in the residuals for N_2_O, while estimating the other components more accurately than before. The results showed an excellent accuracy for all components, with no clear outliers identified and no patterns showing inherent issues with the estimation. This approach was found to be functional and was further improved from changing the solver goal to be based on the relative, instead of the absolute deviations, which allowed for the weighting factors to be equalised for the main components. Weighting factors for secondary components were also reduced to zero for the estimation with the relative difference, the reason being that these components were studied and found not to have realistic conversions in the test data.

Conversion for each component is also equally important to ensure an adequate description of the outlet concentrations over a full-scale catalyst. The final result, however, demonstrated good accuracy over the entire experimental space and with every component. One identified challenge was that the low conversions in the range 0–20% were found to be less accurate. This could be explained by N_2_O formation, which at lower temperatures is poorly described by the model. This could also be attributed to the inaccuracy of the measurements as these observed changes in concentrations were very small for this catalyst type. Therefore, a minor inaccuracy in the measurements could cause deviations in the calculations.

**Fig. 9:**
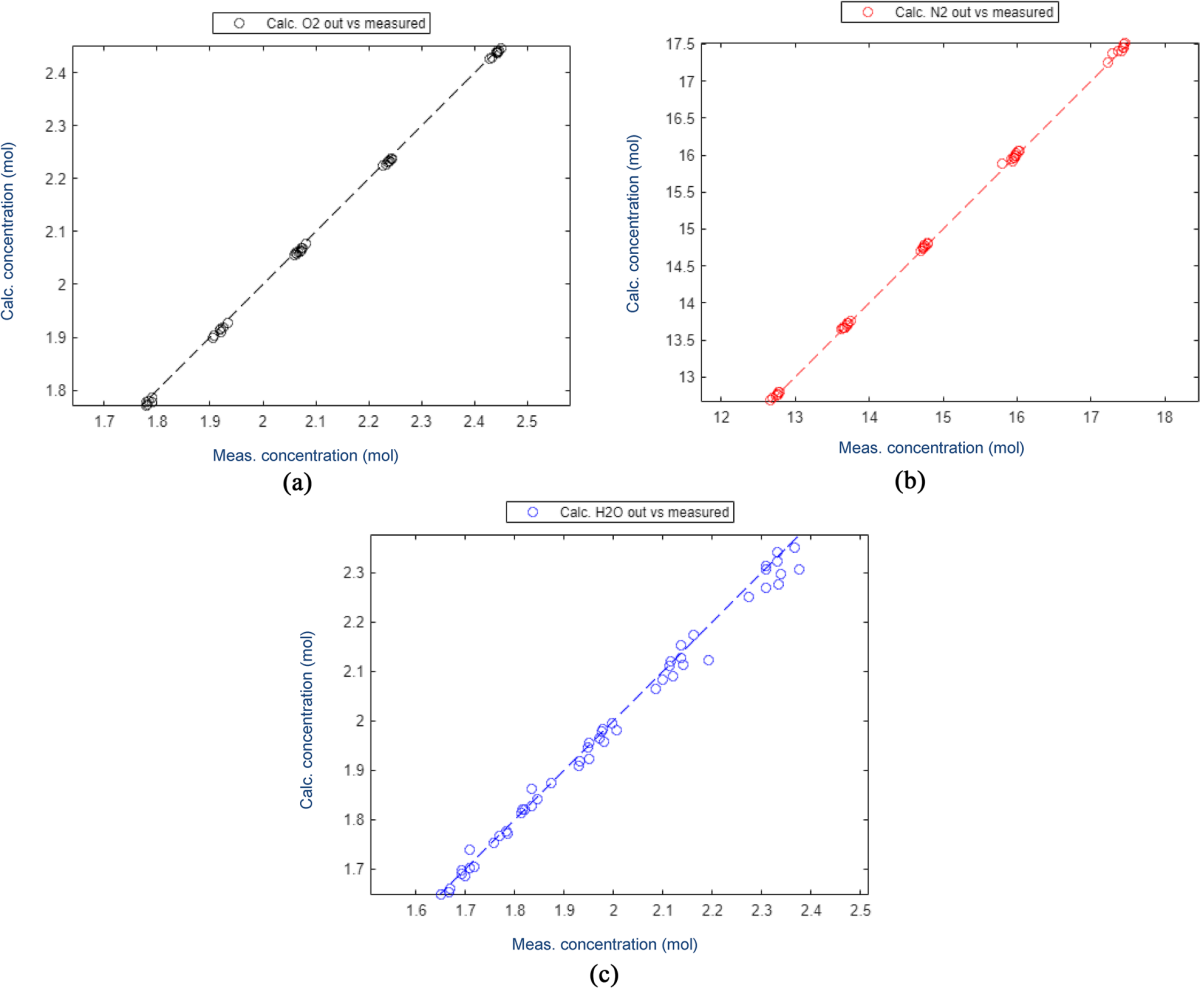
Comparison of estimated and measured concentrations of secondary components for catalyst formation 1.

The components used for validation (Fig. 9) also showed consistent behaviour, although it was determined that changes in the concentrations were several magnitudes smaller than the inlet concentrations and, thus, no apparent deviation would have been observed even with a faulty calculation model. A slight deviation in the water content can also be seen comparing the measured concentrations in the inlet and outlet of the test data, which can be ascribed to inaccuracies in the introduction of water in the feed during testing. Steam was generated using an evaporator and the feed concentration was measured only before adding catalyst to the reactor, as the same analyser was used for the feed compositions as for the measurements of the outlet concentrations.

From the values of the estimated parameters, it is possible to determine how the catalyst is functioning. Comparing the results for catalyst 1 through 3 it can be concluded that the adsorption constants for oxygen and water are very low (Table 3). All other adsorption constants are of a similar magnitude. NO oxidation to NO_2_ seems to be favoured in the forward direction, with the reverse direction, $\:{{\boldsymbol{k}}^{\boldsymbol{a}\boldsymbol{v}\boldsymbol{g}}}_{\boldsymbol{k}3,\boldsymbol{r}}$, being orders of magnitude smaller for all samples than for the forward one. Subsequently this reaction can be treated as irreversible. Furthermore, the undesired oxidation of NH_3_ was found to be small for all samples, suggesting that it does not play a large role in the NH_3_ removal.

**Table 3:** Estimated rate constants at average temperature, relative to k_3f_.

Parameter	Sample 1	Sample 2	Sample 3
${K^{avg}}_{{{O_*}}}$	1.0 × 10^− 3^	2.3 × 10^− 8^	2.3 × 10^− 8^
${K^{avg}}_{{N{O_*}}}$	6.0	5.0 × 10^− 3^	2.5 × 10^− 2^
${K^{avg}}_{{N{O_{2*}}}}$	6.9	7.0 × 10^− 2^	8.7 × 10^− 2^
${K^{avg}}_{{{N_2}{O_*}}}$	2.0	4.8 × 10^− 2^	3.5 × 10^− 2^
${K^{avg}}_{{N{H_{3*}}}}$	1.0	4.0 × 10^− 3^	1.3 × 10^− 2^
${K^{avg}}_{{{H_2}{O_*}}}$	3.0 × 10^− 3^	5.3 × 10^− 11^	6.5 × 10^− 13^
${k^{avg}}_{{k3,f}}$	1.0	1.0	1.0
${k^{avg}}_{{k3,r}}$	0.14	9.6 × 10^− 12^	7.1 × 10^− 4^
${k^{avg}}_{{k6}}$	1.5 × 10^− 2^	6.1 × 10^− 5^	1.9 × 10^− 11^
${k^{avg}}_{{k8}}$	17	6.9 × 10^− 2^	0.38
${k^{avg}}_{{k9}}$	4.0 × 10^− 2^	2.9 × 10^− 13^	3.5 × 10^− 4^
${k^{avg}}_{{k10}}$	2.9	0.18	3.9 × 10^− 2^
${k^{avg}}_{{k11}}$	2.2	4.7 × 10^− 3^	5.5 × 10^− 2^
${k^{avg}}_{{k12}}$	0.96	7.6 × 10^− 4^	3.1 × 10^− 3^
${k^{avg}}_{{k14}}$	10	0.15	0.25
${k^{avg}}_{{k15}}$	3.9	2.8 × 10^− 6^	5.0 × 10^− 15^

From the activation energies (Table 4) it is also possible to determine which reactions are strongly temperature dependent. Furthermore, it can be seen that there are several reactions that are negatively affected by higher temperatures. It is also evident that some reactions are less affected by temperature than others, with activation energies lower than 1.0 kJ/mol. In the best solution found all heats of adsorption are negative, as expected, which allows for the assumption that the adsorption behaviour is in theory correct. However, when comparing the heats of adsorption, no real trends between samples were found as values changed orders of magnitude between the samples. This could be explained by different chemical nature of the tested catalysts. The activation energies of the rate coefficients were more aligned with each other, with only $\:{Ea}_{k9}$, $\:{Ea}_{k12}$ and $\:{Ea}_{k14}$ displaying different behaviour. As the rate constant k_9_ was very low, the activation energy is assumed to have a very low accuracy and a change in magnitude is to be expected between samples.

**Table 4:** Estimated heats of adsorption and activation energies, relative to E_3f_.

Parameter	Sample 1	Sample 2	Sample 3
$\Delta {H_{{O_*}}}$	-1.0	-1.8	-0.80
$\Delta {H_{N{O_*}}}$	-0.21	-2.6 × 10^− 6^	-0.49
$\Delta {H_{N{O_{2*}}}}$	-6.7 × 10^− 3^	-3.4 × 10^− 6^	-3.0 × 10^− 4^
$\Delta {H_{{N_2}{O_*}}}$	-0.11	-4.8 × 10^− 7^	-5.1 × 10^− 2^
$\Delta {H_{N{H_{3*}}}}$	-0.28	-1.7 × 10^− 7^	-0.71
$\Delta {H_{{H_2}{O_*}}}$	-7.6 × 10^− 6^	-2.9 × 10^− 2^	-3.6 × 10^− 5^
$E{a_{k3,f}}$	1.0	1.0	1.0
$E{a_{k3,r}}$	0.36	0.56	0.38
$E{a_{k6}}$	0.76	1.4	0.24
$E{a_{k8}}$	-6.4 × 10^− 2^	-0.15	-3.5 × 10^− 2^
$E{a_{k9}}$	0.10	0.19	-0.24
$E{a_{k10}}$	-0.12	-1.8 × 10^− 2^	-0.12
$E{a_{k11}}$	0.28	0.53	0.34
$E{a_{k12}}$	-0.29	0.25	0.26
$E{a_{k14}}$	0.28	0.22	0.58
$E{a_{k15}}$	-2.0	-0.44	4.3

Utilization of a large number of parameters to be fitted inevitably raises a question of over -parameterization and potential correlation between the parameters. To address this point sensitivity analysis was performed using the Markov Chain Monte Carlo method^18^. This method was incorporated in the modelling and optimization software ModEst^19^, allowing evaluation of the reliability of the model parameters by treating all the uncertainties in the data and in the modelling as statistical distributions^20^.

**Fig. 10:**
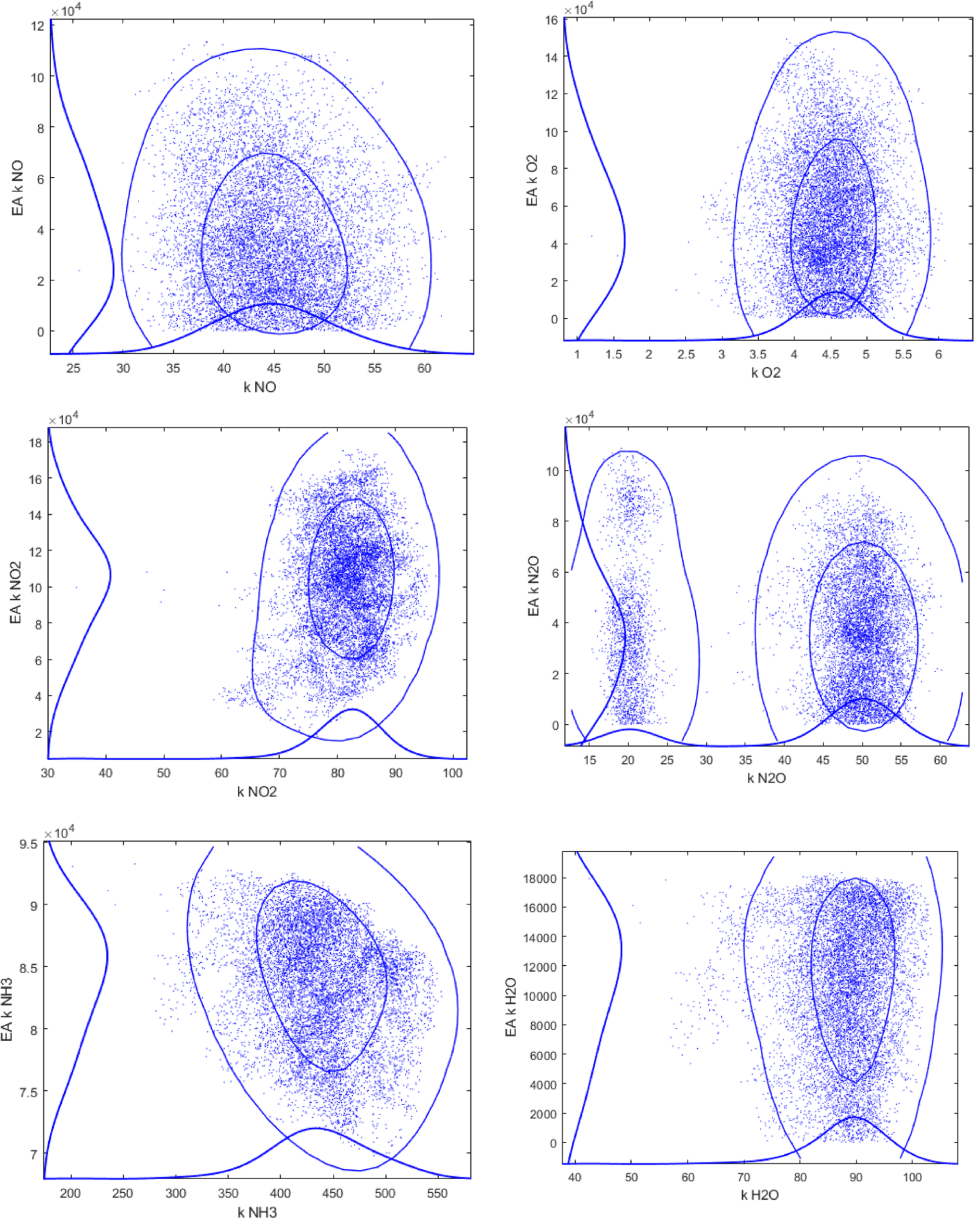

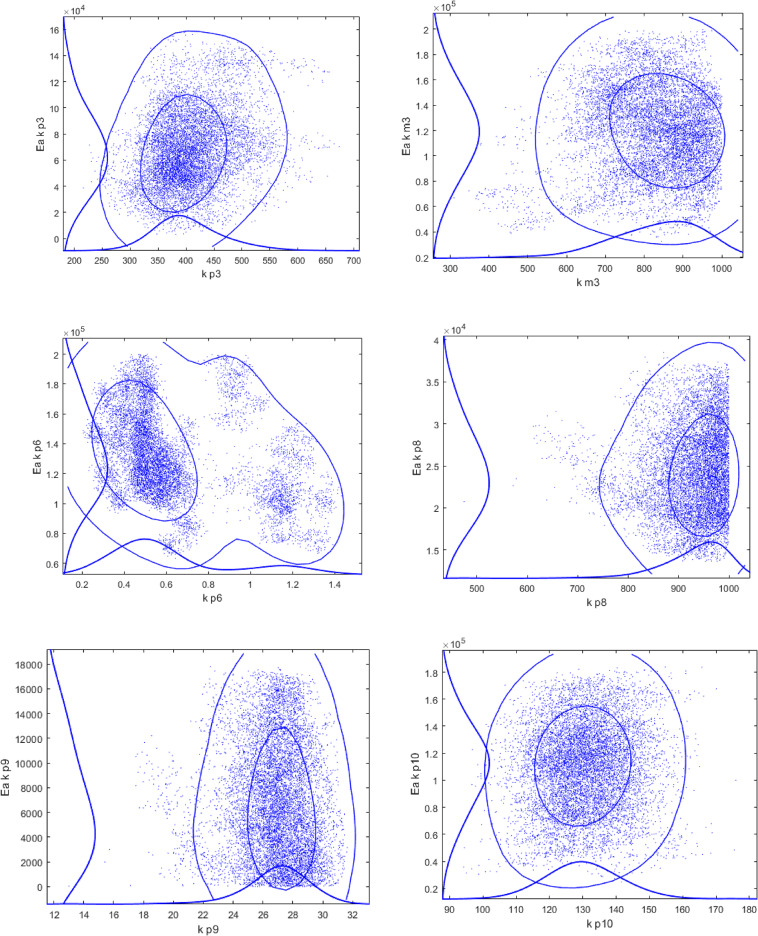

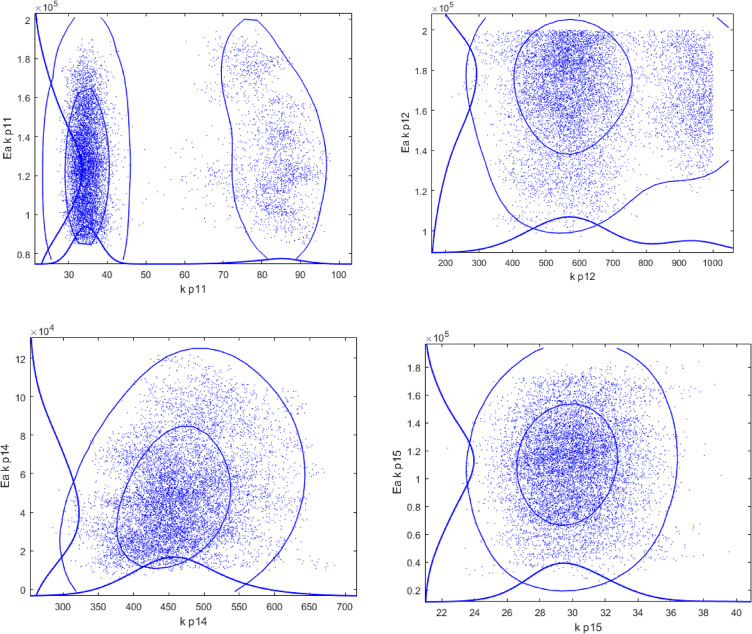
Monte Carlo Markov Chain analysis for pairs of parameters for sample 1.

The results of the sensitivity analysis, presented in Fig. 10, illustrate that the values of kinetic parameters for the majority of pairs are rather well defined.

### Full scale verification

The model was verified in full scale tests for a real marine application. More specifically a marine NH_3_ engine with a SCR system was tested with the same catalyst as one of the samples.

The model, with the constants fitted to the small-scale samples, was used to calculate the outlet concentrations from the inlet conditions during full scale testing, and later compared to actual test measurements.

**Fig. 11:**
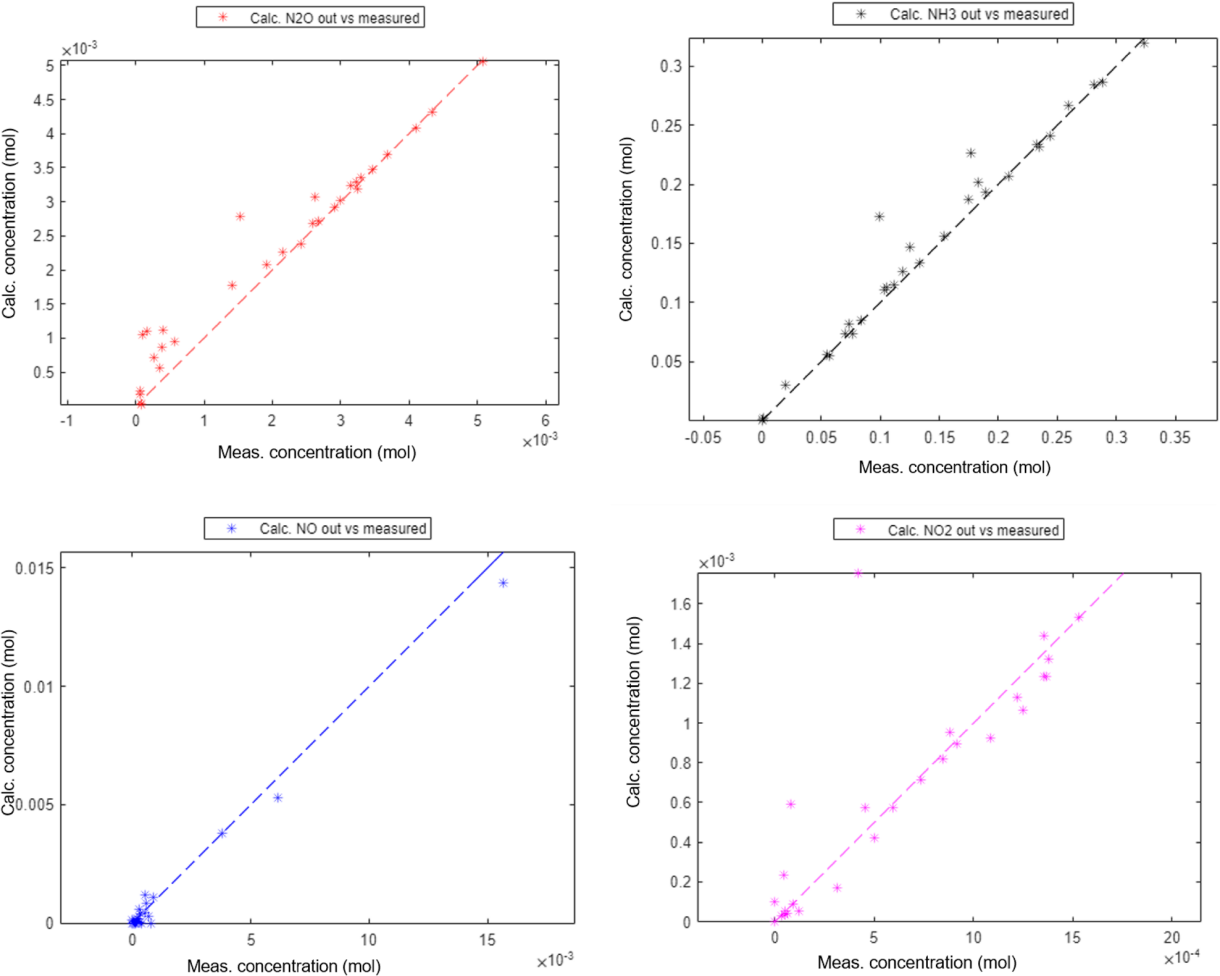
Comparison between measured and calculated concentrations in full scale verification tests.

Looking at the absolute concentrations, the model was exhibiting a good accuracy to the measured concentrations of the components. The catalyst was not exposed to high NOx concentrations in the tested operational areas at full scale, showing nevertheless good accuracy. The results are presented in Fig. 11 for concentrations of N_2_O, NH_3_, NO and NO_2_.

When comparing conversion of N_2_O and NH_3_ there was an apparent difference between the kinetic model estimations and the measured data for some data points. Interestingly enough, the catalyst seemed to be performing better in the full-scale version than it had performed in the lab reactor testing. Noteworthy is, that the concentrations of NH_3_ were higher than that of the test data used for parameter estimation which, in practice, means that there might be errors in the estimated parameters that could explain the lab scale data with a limited extrapolation ability. This was assumed to be the cause of the errors. The precision for calculations of NO and NO_2_ conversion is heavily dependent on very low concentrations of both components being, most likely, affected by measuring accuracy of applied analytical method (FTIR) rather than predictive ability of the kinetic model.

## Conclusions

For this work, lab scale testing of a catalyst for N_2_O removal and the subsequent model development was done in an effort to describe the kinetics of similar catalysts in exhaust aftertreatment. This was followed by a full scale verification with an ammonia engine.

The emphasis was placed on analysis of a generic kinetic model and illustrating how the model can be used for describing a particular set of data with commercial catalysts, when the exact composition cannot be revealed.

Nevertheless, from the analysis of the test data, correlations between the behaviour of certain components and the underlying mechanisms were brought together in a combination with what had been discussed in the literature to model the complex reactions taking place on the surface of the catalyst. The developed model was also checked against the thermodynamics of the proposed reactions and the reactivity of the gaseous components in the specified operational window.

The kinetic model was successful in describing the behaviour of catalyst formulations. The model is detailed enough to be used in combination with other models to determine the functionality of the catalyst in a commercial application if the required parameters and measurements are available or can be assumed to be of sufficient accuracy.

In this work, the kinetic model has been built on the assumption that all components adsorbed on the surface of the catalyst are competing for the same active sites. This was assumed from inhibition for almost all components upon increasing concentrations of ammonia or water. Most kinetic models for N_2_O catalyst have used only NH_3_ as an adsorbed component, while all other components have been supposed to react from the gas phase. This simplification can be done if the adsorption coefficients for all other components are very low, although, as this model was built fully from the start, a complete model with all coverages was used. The use of different active sites provides a possibility to have a better description of a certain behaviour.

Utilization of a residence time-dependent kinetic model introduces a minor inaccuracy as the residence time is calculated from the volumetric flow of the exhaust gas and the catalyst bed dimensions. The model uses a mean catalyst temperature to calculate the volumetric flows of the exhaust. In a real catalyst bed, the temperature would increase from the released heat of the reactions taking place on the catalyst surface. Ammonia oxidation and NOx reduction are both highly exothermic reactions and have positive temperature dependency of the rates. At the same time, the model only uses the total molar flow at the inlet of the catalyst. This introduces yet another inaccuracy, as most reactions taking place on the surface of the catalyst are net positive as per respect to the total molar flow. This was investigated and found to be smaller than 1%, but it would still cause the residence time to be decreased.

To increase accuracy of the model it would be beneficial to estimate the parameters on a larger set of test data. This would allow for outliers to be identified as they would not be explained by the model at all, and could, thus, be left out of the data set. The issue with the available data was that there were not enough data points for accurate determination of parameters. Preferably, there should be a minimum of three data points in close proximity of each other for each operating condition. This would allow for excluding points found to be outliers. Because of this, the model should be used for preliminary evaluation of the catalyst performance.

The developed model, however, gives a possibility to determine catalytic performance under operating conditions beyond the tested ones. Such information avoids a need for full testing over a complete range of operating conditions. Practical application for the developed approach is in the emission aftertreatment systems for ammonia engines, which due to its immature character, might not have the best possible operating profiles yet. Whenever a new operating profile for such an engine would be developed, this model would allow for testing of what the N_2_O removal catalyst will achieve under new operating conditions.

## Supplementary Information

Below is the link to the electronic supplementary material.

Supplementary Material 1

## Data Availability

The datasets used and/or analysed during the current study available from the corresponding author on reasonable request.
