# Supplementary material for: Kinetic modelling of catalytic N2O removal
Source: Sci Rep. 2025 Dec 29;15:44959. doi: 10.1038/s41598-025-28647-6 (PMC12750005; doi:10.1038/s41598-025-28647-6)

Kinetic modelling of catalytic N_2O removal

Mathias Nygård[1,2], Johan Wärnå[1], Vincenzo Russo[3],
Jan Torrkulla[2], Dmitry Yu. Murzin[1*]

[1]Åbo Akademi University, Henriksgatan 2, 20500, Åbo, Finland
[2]Wärtsilä Finland Oy, FI-65101, Vaasa, Finland
[3]Università di Napoli 'Federico II', Chemical Sciences, IT-80126 Napoli, Italy

Supporting Information

Comparision of models

Comparison between the model in Table 1 of the article (A) with the alternative model (B) having an extra step **N2O+O* -> 2NO**

The parameter estimations for models A and B have ended up at practically same degree of explanation 97.87% and 97.85%. For model B the estimated rate constant for the additional reaction is $0.196 \cdot 10^{-06}$, being practically equal to zero. From the graphs only a very small difference in the fits between the models is visible (Model A blue dots and model B red +) .

Catalyst 1

Model A

Explained (%): 97.87

Model B, with the additional reaction 16 N2O+O* -> 2NO

Explained (%): 97.85

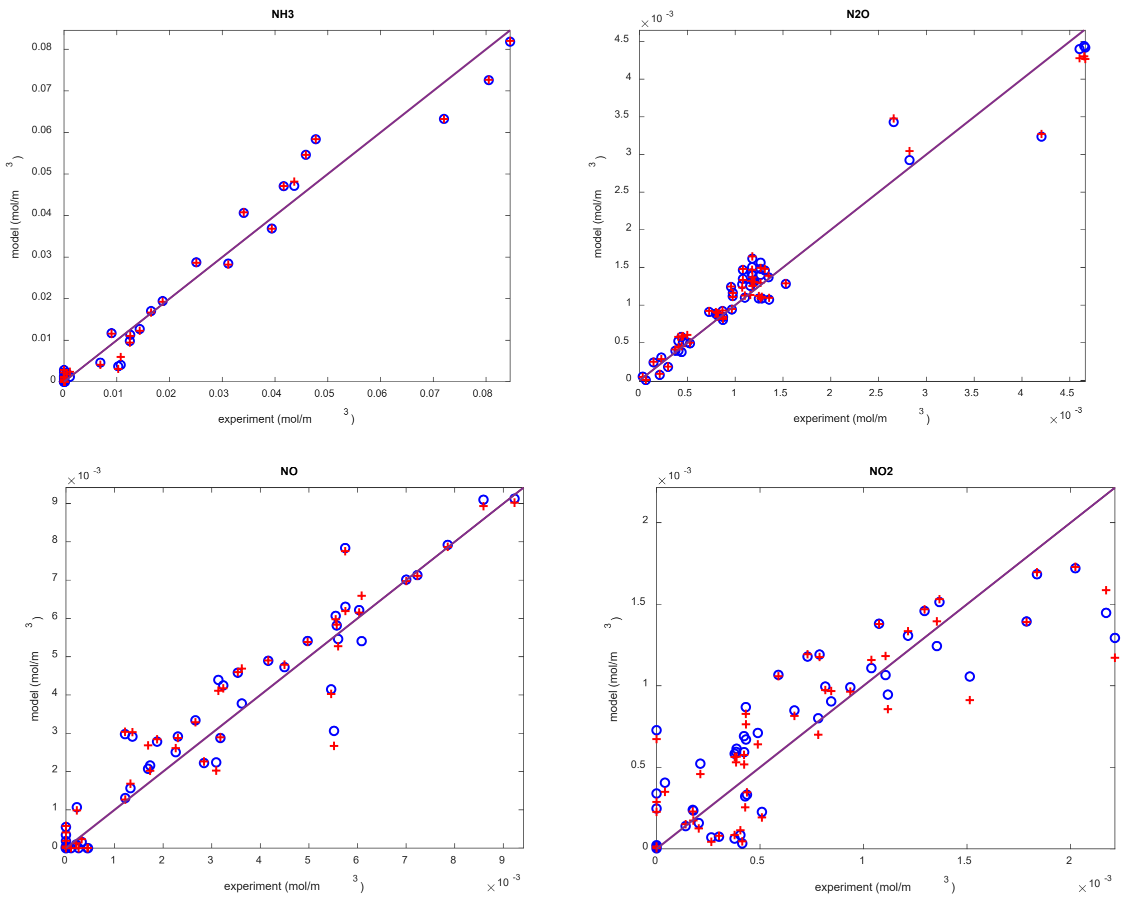

Catalyst 2

Model A

Explained (%): 98.69

Model B, added the additional reaction 16 N2O+O* -> 2NO

Explained (%): 98.92

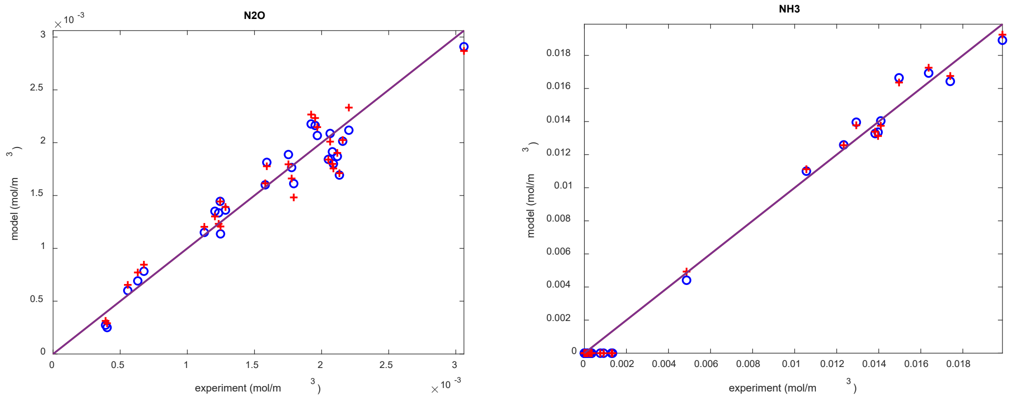

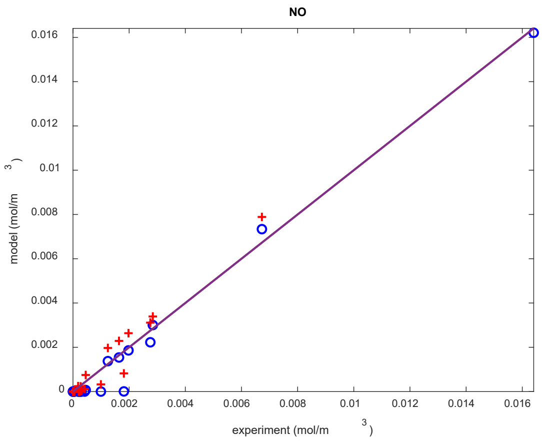

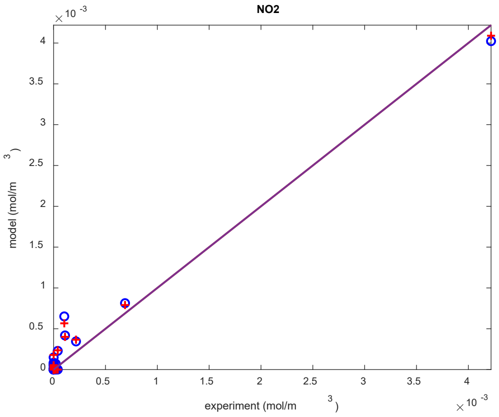

Catalyst 3

Model A

Explained (%): 98.69

Model B, added the additional reaction 16 N2O+O* -> 2NO

Explained (%): 98.70

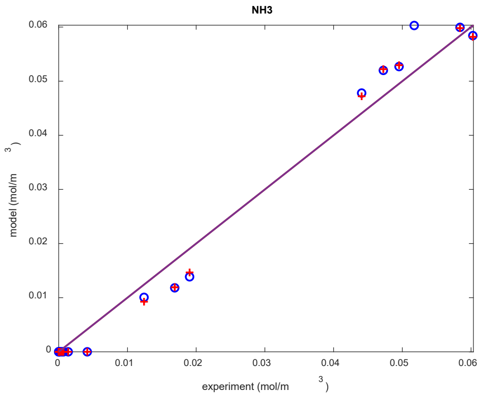

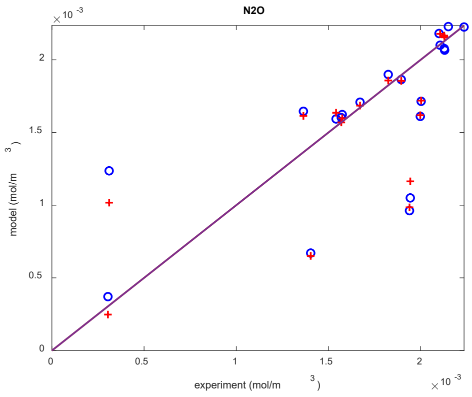

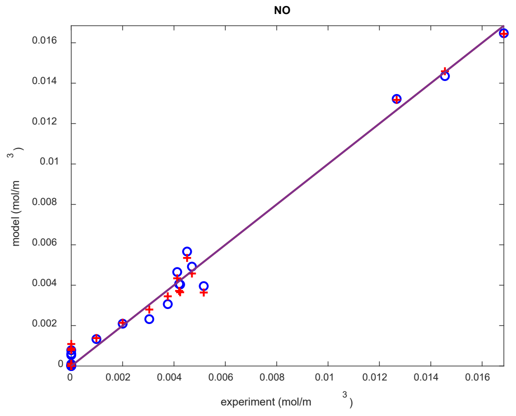

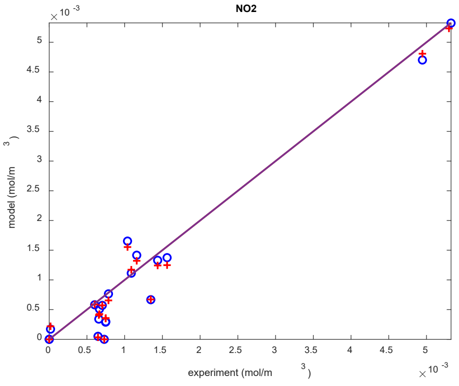

Supplement: Supplementary file 1 — Supplementary Material 1 [file 41598_2025_28647_MOESM1_ESM.pdf]
